# 1-Aminocyclopropanecarboxylic acid (ACPC) produces procognitive but not antipsychotic-like effects in rats

**DOI:** 10.1007/s00213-014-3738-4

**Published:** 2014-09-27

**Authors:** Piotr Popik, Malgorzata Holuj, Agnieszka Nikiforuk, Tomasz Kos, Ramon Trullas, Phil Skolnick

**Affiliations:** 1 Behavioral Neuroscience and Drug Development, Institute of Pharmacology, Polish Academy of Sciences, 12 Smetna Street, 31-343 Kraków, Poland; 2 Institut d’Investigacions Biomèdiques August Pi i Sunyer, IDIBAPS-CSIC, 08036 Barcelona, Spain; 3 Division of Pharmacotherapies & Medical Consequences of Drug Abuse, NIDA, NIH, Bethesda, MD 20892-9551 USA

**Keywords:** 1-Aminocyclopropanecarboxylic acid, ACPC, Strychnine-insensitive glycine/NMDA site, Learning, Memory, Cognitive flexibility, Attentional set-shifting test, Schizophrenia, Animal models, Prefrontal cortex, Attention, Impulsivity, Five-choice serial reaction time task (5-CSRTT)

## Abstract

**Rationale:**

In addition to the negative and positive symptoms of schizophrenia, cognitive deficits, including prefrontal cortical dysfunction, are now recognized as core features of this disorder. Compounds increasing the NMDA receptor function via the strychnine-insensitive glycine receptors have been proposed as potential antipsychotics. Depending on the ambient concentrations of glutamate and glycine, 1-aminocyclopropanecarboxylic acid (ACPC) behaves as either a partial agonist or a functional antagonist at the strychnine-insensitive glycine receptors.

**Objectives:**

We investigated the procognitive and antipsychotic-like effects of ACPC in rats treated with phencyclidine (PCP) or ketamine (KET), compounds that produce psychotic-like symptoms in humans and laboratory animals.

**Methods:**

Cognitive effects were investigated in the novel object recognition (NOR) and attentional set-shifting tests (ASST). In addition, the effects of ACPC were investigated in PCP-induced hyperactivity, conditioned avoidance response (CAR), and prepulse inhibition (PPI) tests. The effects on attention and impulsivity were measured in the five-choice serial reaction time task (5-CSRTT).

**Results:**

ACPC (200–400 mg/kg) inhibited memory fading in naive rats and like clozapine prevented PCP- and KET-induced amnesia in the NOR. In naive animals, ACPC at 400 but not 200 mg/kg enhanced cognitive flexibility in the ASST, as the animals required fewer trials to reach the criteria during the extra-dimensional phase. In contrast, ACPC did not affect PCP-induced hyperactivity, CAR, and PPI as well as attention and impulsivity in the 5-CSRTT.

**Conclusion:**

The present study demonstrates that ACPC enhanced both object recognition memory and cognitive flexibility dependent on the prefrontal cortex, but did not affect impulsivity nor exhibit an antipsychotic-like profile.

## Introduction

Glutamatergic transmission through the *N*-methyl-d-aspartate receptor (NMDAR) receptor subtype plays a critical role in neuroplasticity. Furthermore, it has been suggested that an increase in NMDAR activity may lead to cognitive enhancement (Danysz and Parsons 1998). Augmentation of glutamatergic neurotransmission may also produce antipsychotic effects; for a review, see Hashimoto et al. (2013). Conversely, NMDA antagonists such as phencyclidine (PCP) and ketamine (KET) elicit positive, negative, and cognitive psychotic-like symptoms in normal volunteers and produce a recrudescence of psychotic symptomatology in patients (Javitt and Zukin 1991; Krystal et al. 1994; Jentsch and Roth 1999).

Activation of the NMDAR requires binding of both glutamate and its co-agonist, glycine, a small nonessential amino acid (Johnson and Ascher 1987). Since an enhancement of glutamatergic tone by direct (orthosteric) NMDA receptor agonists does not appear to be a feasible therapeutic strategy, a more practical approach is to enhance the function of strychnine-insensitive glycine sites. This can be done using full and partial glycine agonists or by enhancing glycine availability at the synaptic cleft by inhibiting reuptake through glycine transporter 1 (GlyT1; (Raiteri and Raiteri 2010)). The rationale for this approach relies on the assumption that strychnine-insensitive glycine sites at the NMDAR are not saturated under physiological conditions (for a review, see (Danysz and Parsons 1998)).

Consistent with this approach, both antipsychotic and procognitive effects have been demonstrated for compounds increasing transmission at glycine sites. Almost 30 years ago, glycine itself was shown to reduce PCP-induced hyperactivity in mice (Toth and Lajtha 1986). GlyT1 inhibitors that elevate ambient glycine concentrations reversed or attenuated NMDAR antagonist-induced abnormally persistent latent inhibitions (Lipina et al. 2005; Black et al. 2009) and deficits of prepulse inhibition (PPI) (Lipina et al. 2005; Depoortere et al. 2005; Boulay et al. 2008). In the latter paradigm, the NMDA receptor antagonist ketamine dose-dependently reduces PPI (Swerdlow et al. 1998). While the prototypic antipsychotic haloperidol is inactive per se and does not reverse ketamine-induced reduction of PPI, chlorpromazine, seroquel, and clozapine are active in this model. In general, reversal of NMDA antagonist-induced decreases in PPI is considered an antipsychotic-like effect (see Geyer et al. (2001) for the review).

Other studies demonstrated that D-serine, a full agonist at the strychnine-insensitive glycine sites, enhanced social memory in rats (Shimazaki et al. 2010) and that D-cycloserine, a partial agonist at the strychnine-insensitive glycine sites, facilitated learning and memory in rats (Monahan et al. 1989). Inhibitors of GlyT1 have also demonstrated procognitive effects in preclinical models of schizophrenia. For instance, SSR-103800 reversed PCP-induced impairment of short-term episodic-like memory in the object recognition task (Boulay et al. 2008), whereas NSFP improved dizocilpine (MK-801)-disrupted social memory (Shimazaki et al. 2010). Recent data from this laboratory (Nikiforuk et al. 2011) demonstrated that SSR-504734, a non-sarcosine GlyT1 inhibitor, enhanced cognitive performance in the attentional set-shifting test (ASST), a measure of prefrontal flexibility that is compromised in schizophrenic patients (Lewis and Gonzalez-Burgos 2008) as well as in healthy volunteers (Krystal et al. 1994) and rats treated with NMDAR antagonists (Stefani et al. 2003; Egerton et al. 2005; Nikiforuk et al. 2010).

In clinical studies, glycine (Javitt et al. 1994), D-serine, and sarcosine have been shown to ameliorate symptoms of schizophrenia in medicated patients (Lane et al. 2005; Tsai and Lin 2010). Meta-analyses indicate that glycine, D-serine, and sarcosine are more effective than D-cycloserine in improving the overall psychopathology in schizophrenic patients receiving antipsychotic drugs (Heresco-Levy and Javitt 2004; Tuominen et al. 2005; Tsai and Lin 2010), suggesting a relatively narrow therapeutic window for partial agonists at the glycine sites (Hashimoto et al. 2013).

1-Aminocyclopropanecarboxylic acid (ACPC) is a high-affinity partial agonist at the strychnine-insensitive glycine sites (Marvizon et al. 1989) that exhibits anticonvulsant (Witkin and Tortella 1991), neuroprotective (Von Lubitz et al. 1992; Fossom et al. 1995), anxiolytic (Trullas et al. 1989), anti-addictive (Kolesnikov et al. 1994) and antidepressant-like (Trullas and Skolnick 1990; Trullas et al. 1991; Papp and Moryl 1996) effects in rats and mice. This broad range of pharmacological effects is reminiscent of NMDA antagonists.

However, in contrast to NMDA receptor antagonists acting at other loci and the full antagonist of the glycine site 7-chloro-kynurenic acid that impair spatial learning (Bannerman et al. 1997; Carli et al. 2001), the functional NMDA/glycine receptor antagonist ACPC does not impair memory or learning processes in a step-through avoidance (Faiman et al. 1994). Conversely, preclinical studies demonstrated that ACPC facilitates spatial learning in senescent rats (Popik and Rygielska 1999) and in mice tested in a single-trial inhibitory avoidance learning both in naive animals and following administration of a variety of amnestic agents (Viu et al. 2000). These properties make ACPC a particularly interesting molecule to investigate both in tests addressing cognitive deficits observed in schizophrenia (NMDAR antagonist-disturbed conditions, prefrontal cognitive flexibility) as well as in procedures used to detect potential antipsychotic activity (PCP-induced hyperactivity, CAR, PPI). Here, ACPC was also tested in the five-choice serial reaction time task (5-CSRTT), a procedure that permits the simultaneous examination of multiple aspects of attentional performance (Bari et al. 2008) including the accuracy of attentional processes and impulsivity. While NMDAR antagonists (e.g., MK-801, PCP) have been demonstrated to disrupt attentional performance and/or increase impulsivity (reviewed in (Amitai and Markou 2010)), the effects of glycinergic compounds have not been widely assessed in this task.

## Materials and methods

### Animals

Male Sprague-Dawley rats (Charles River, Germany) weighing 250–280 g on arrival were used in this study. Animals were initially group-housed (five rats/cage) in a temperature- (21 ± 1 °C) and humidity- (40–50 %) controlled colony room under a 12/12-h light/dark cycle (lights on at 06:00 h). Rats were allowed to acclimatize for at least 7 days before the start of the experimental procedures. In the ASST, rats were individually housed with mild food restriction (15 g of food pellets per day) and ad libitum access to water for 1 week prior to testing. For the 5-CSRTT, rats were group-housed (four rats/cage) with mild food restriction (15 g of food pellets per day). Behavioral testing was performed during the light phase of the light/dark cycle. The experiments were conducted in accordance with the NIH Guide for the Care and Use of Laboratory Animals and were approved by the Ethics Committee for Animal Experiments, Institute of Pharmacology. All rats were used only once, and each experiment was carried out on a separate cohort of animals.

### Novel object recognition test (NOR)

The protocol described earlier (Nikiforuk et al. 2013) was adapted from the original work of Ennaceur and Delacour (1988). At least 1 h before the start of the experiment, the rats were transferred to the experimental room for acclimation. Animals were tested in a dimly lit (25 lx) open-field apparatus made of a dull gray plastic (66 × 56 × 30 cm). After each measurement, the floor was cleaned and dried. The procedure consisted of a 5-min habituation to the arena without any objects, 24 h before the test. The testing comprised two trials separated by an inter-trial interval (ITI). To test the memory improvement in naive animals, an ITI of 24 h was chosen. To test the memory improvement in either PCP- or KET-disturbed conditions, a 1-h ITI was chosen.

During the first (familiarization, T1) test period, two identical objects (A1 and A2) were presented in the opposite corners of the arena, approximately 10 cm from the walls. Following T1, the objects were cleaned with water containing a dishwashing agent and dried. In the second trial (recognition, T2) one of the objects was replaced by a novel one (A = familiar and B = novel). Both trials lasted for 3 min. After T1, animals were returned to their home cages. The objects used were a 250-ml glass beaker (diameter of 8 cm, height of 14 cm) filled with gravel (350 g) and a 250-ml plastic bottle (6 × 6 × 13 cm) filled with sand (450 g). The location of the novel object in T2 was randomly assigned for each rat. Exploration of an object was defined by rats looking, licking, sniffing, or touching the object, but not leaning against, standing, or sitting on the object. The exploration time of the objects was measured using the Any-maze® tracking system (Stoelting Co., IL, USA). Based on the exploration time (*E*) of the two objects, a discrimination index was calculated in accordance with the formula discrimination index (DI) = (*E*_B_–*E*_A_)/(*E*_A_ + *E*_B_), where *E*_A_ is defined as the time spent exploring the familiar object and *E*_B_ is the time spent exploring the novel object, respectively.

### Attentional set-shifting test (ASST)

Testing was conducted in a modified wire rat housing cage (42 × 32 × 22 cm) with a white plywood wall dividing half of the length of the cage into two sections. During testing, one ceramic digging pot (diameter of 10 cm, height of 2 cm) was placed in each section. Each pot was defined by a pair of cues along with two stimulus dimensions (the digging media and odors, as described in details by Nikiforuk et al. (2010)). To mark each pot with a distinct odor, 5 μl of a flavoring essence (Dr. Oetker®, Poland) was applied to a small piece of blotting paper fixed to the external rim of the pot immediately prior to use. A different pot was used for each combination of digging medium and odor; only one odor was ever applied to a given pot. The bait (one-third of a Honey Nut Cheerio, Nestle®) was placed at the bottom of the “positive” pot and buried in the digging medium.

The procedure was adapted from Birrell and Brown (2000) and entailed three testing days for each rat, as described in details elsewhere (Nikiforuk et al. 2011). In simple discrimination (SD) involving only one stimulus dimension, the pots differed along one of the two dimensions (i.e., a digging medium). For compound discrimination (CD), a second, irrelevant, dimension (i.e., an odor) was introduced, but the correct and incorrect exemplars of the relevant dimension remained constant. For the reversal of this discrimination (Rev 1), the exemplars and relevant dimension were unchanged, but the previously correct exemplar was now incorrect and vice versa. The intra-dimensional (ID) shift was then presented, comprising new exemplars of both the relevant and irrelevant dimensions with the relevant dimension remaining the same as previously. The ID discrimination was then reversed (Rev 2) so that the formerly positive exemplar became the negative one. For the most essential, extra-dimensional (ED) shift, a new pair of exemplars was again introduced, but this time, a relevant dimension was also changed. Finally, the last phase was the reversal (Rev 3) of the ED discrimination problem. The exemplars were always presented in pairs and varied so that only one animal within each treatment group received the same combination. The following pairs of exemplars were used: pair 1: odor: lemon vs. almond, medium: cotton wool vs. crumpled tissue; pair 2: odor: spicy vs. vanilla, medium: metallic filler vs. shredded paper; and pair 3: odor: rum vs. cream, medium: clay pellets vs. silk. Our previous study demonstrated that there were no differences in the performance of rats shifted from odor to medium and from medium to odor (Nikiforuk et al. 2010). Therefore, in an attempt to simplify the experimental design, the order of discrimination was always the same (i.e., from digging medium to odor). The assignment of each exemplar in a pair as being positive or negative at a given phase and the left-right positioning of the pots in the test apparatus on each trial were randomized.

### Spontaneous and PCP-induced hyperactivity

Spontaneous and PCP-induced locomotor activity was measured automatically in Opto-Varimex-4 Auto-Tracks (Columbus Instruments, OH, USA) located in sound-attenuated and ventilated boxes. The Auto-Track System sensed the motion with a grid of infrared photocells (16 beams per axis) surrounding the arena.

### Conditioned avoidance response (CAR)

The training and the testing were performed in four shuttle boxes (Med Associates, Inc., USA). Each box (44 × 21 × 18 cm) was housed in ventilated, sound-isolated cubicles and was divided in two equal-sized compartments by guillotine doors. The rats were allowed to move freely from one compartment to another at any time. The position of the animal was tracked by eight photocells in each of the boxes. A cue light was situated on the wall opposing the compartment entry.

The sessions were started by presenting the conditioned stimulus (CS; the light) for 10 s, followed by an unconditioned stimulus (UCS; continuous foot shock of 0.37 mA) for the maximum duration of 10 s. The procedure was repeated with 20 trials daily with an intra-trial interval of 20–40 s. If a rat moved from one compartment to another within 10 s of CS presentation, it avoided the foot shock, and this shuttle response was recorded as *avoidance*. If the rat remained in the compartment for more than 10 s and made a crossing upon receiving the foot shock, this response was recorded as an *escape.* If the rats did not respond either during the 10 s of CS or by 10 s of UCS, the trial was terminated and escape *failure* was recorded. It is known that compounds displaying antipsychotic activity selectively inhibit avoidance response without affecting escapes and failures (Wadenberg and Hicks 1999).

About 12–15 training sessions, lasting for 2–3 weeks, were needed to train the animals to the stable avoidance level of above 80 % in two consecutive days. The rats fulfilling these criteria (~60 %) were given different doses of ACPC or clozapine as a positive control. Due to the nature of the test, allowing for repeated testing (Wadenberg and Hicks 1999), animals were drug tested up to three times with a 7-day drug-free period between tests according to a randomized design (Wadenberg et al. 1997).

### Prepulse inhibition of the acoustic startle response (PPI)

The PPI procedure was adopted from a published protocol (Auclair et al. 2006). Rats were subjected to two pretest sessions: an afternoon session on the day before testing and a morning session on the test day (Fijal et al. 2014). We used a startle apparatus (Med Associates, Inc., USA) consisting of acrylic animal holders with a grid floor made of stainless bars, mounted onto a startle platform placed in a ventilated, sound-attenuated chambers (Nikiforuk et al. 2013). Acoustic stimuli were generated by two speakers: a background-noise speaker and a stimulus speaker, placed at the back of the chamber, 7 cm from an animal holder. Startle responses were detected and transduced by the load cell, then digitized and stored by Startle Reflex Software (Med Associates, version 5).

Each session started with a 5-min acclimatization period. A 62-dB background white noise was continuously presented once animals were placed in the test chambers. The following types of acoustic stimuli were used in the test protocol: pulse alone [intensity, 120 dB; duration, 40 ms, (P)], pulse preceded by an acoustic prepulse of intensities 70, 73, and 76 dB [duration, 20 ms, (PP)], applied 100 ms before the pulse (P), prepulse alone [intensities, 70, 73, and 76 dB; duration, 20 ms], and a null period. The session consisted of three blocks. During the first block, the animals were exposed to ten pulse-alone trials. During the second block, the following trials were presented in random order: pulse alone, pulse preceded by each prepulse, along with one repetition of each prepulse alone, and four null trials. The inter-trial interval was 20 s. The third block consisted of ten pulse-alone trials. Earlier experiments in this laboratory demonstrated that four presentations of each trial type—as compared to the more common 10 presentations protocol—consistently revealed the antipsychotic-like activity of clozapine (5 mg/kg IP, given 25 min before the test (Fijal et al. 2014)). Therefore, in the present experiments, we used four repetitions of each trial type.

### Five-choice serial reaction time task (5-CSRTT)

Two separate cohorts were trained in the 5-CSRTT. Eight 5-CSRTT operant chambers (Med Associates, Inc., USA), measuring 56 × 56 × 40.5 cm, were housed in sound-attenuated, ventilated cubicles. In each chamber, an array of five square nose-poke holes (2.5 × 2.5 × 2.5 cm) was arranged on a curved panel and raised 2.5 cm from the grid floor. Each hole was equipped with an infrared detector and a yellow stimulus light at its rear. The food magazine, equipped with photocell beams and light, was located on the opposite wall. Food pellets (45 mg, Bio-Serv, USA) were delivered via a dispenser connected to the food magazine. A house light was located 17 cm above the top edge of the food magazine. Online control of the apparatus and data collection was performed using MED-PC (Med Associates, Inc., USA).

In the initial training phase of magazine training, rats had to learn that food pellets were available in the magazine. On the first day, rats were habituated to the operant chambers for 15 min. During this habituation session, the food magazine was filled with several pellets. Next, rats were given magazine training sessions, in which every head entry into a food magazine resulted in a pellet delivery. Once all rats ate 100 food pellets within a session (which usually took one–two sessions), the training proceeded to the next stage.

In the next phase of pretraining, rats had to learn to associate a nose-poke response into an illuminated hole with a pellet delivery. All of the five holes were illuminated, and a response in any aperture was rewarded by a food pellet. This procedure was continued daily until rats obtained 100 pellets within a session.

In the 5-CSRTT training and testing phase, each session began with the illumination of the house light and delivery of a food pellet. A nose poke into the magazine tray initiated the first trial, which consisted of an inter-trial interval (ITI) followed by the random illumination of one of the five holes for a fixed interval (stimulus duration, Sd). If a nose poke was registered in the illuminated hole before the end of the limited hold (Lho, i.e., a fixed interval after Sd), a pellet was delivered and a correct trial was registered. An incorrect response or a failure to respond within the required period (omission) resulted in a time-out (TO) period, in which the house light was extinguished. Responding to any of the five holes during the ITI (premature response) also resulted in a TO. For the first session of training, the stimulus duration and limited hold periods were both set at 60 s and the ITI and TO were 2 s. These variables were gradually altered during training, so the final test parameters were as follows: Sd = 1 s, Lho = 5 s, ITI = 5 s, TO = 5 s. Rats had been trained until they reached the criteria of accuracy >70 %, omissions <30 %, and stable baseline performance over five consecutive sessions (that took approximately 60 training sessions). Each session lasted for 30 min or until 100 trials had been completed. During testing sessions, all rats completed 100 trials within 30 min.

### Drugs

1-Aminocyclopropanecarboxylic acid (ACPC) kindly donated by Dr. M-L Maccecchini, phencyclidine hydrochloride (Sigma-Aldrich, USA), ketamine (aqueous solution (115.34 mg/ml; Biowet Pulawy, Poland)), and MK-801 maleate (Abcam Biochemicals, Cambridge, UK) were dissolved in distilled water. Clozapine (Abcam Biochemicals, Cambridge, UK) was dissolved in 0.1 N HCl supplemented with distilled water to the appropriate volume (final pH = 5.0–6.0). All compounds were administrated in a volume of 1 ml/kg, except ACPC at 400 mg/kg which was administered in a volume of 2 ml/kg due to solubility limitations.

### Drug administration

The doses, routes, and times of administration of compounds that maximized the chance to observe a given effect were selected based on previously published data (Popik and Rygielska 1999; Nikiforuk et al. 2010, 2011, 2013; Fijal et al. 2014).

#### Novel object recognition test (NOR)

In the naive animals, ACPC was administered IP, 30 min before familiarization phase (T1). Separate groups of animals tested in the NMDAR antagonist-disturbed conditions, received ketamine (20 mg/kg, IP) and phencyclidine (5 mg/kg, IP) 45 min before T1, and either ACPC or clozapine was administered 30 min before ketamine, PCP, or vehicle.

#### Attentional set-shifting test (ASST)

ACPC (200–400 mg/kg IP) was administered 30 min before the test.

#### Spontaneous and PCP-induced hyperactivity

Separate groups of animals were administered ACPC, clozapine, or their vehicles IP, 30 min before being placed individually into the auto-tracks for 30 min of spontaneous locomotor activity measurement. Thereafter, the same rats were removed from the boxes and injected with PCP at a dose of 5 mg/kg (SC), and then, the PCP-induced locomotor activity was measured for the following 15–45 min.

#### Conditioned avoidance response (CAR)

ACPC (200–400 mg/kg IP) and clozapine (1–6 mg/kg, IP) were administered 30 and 60 min before the test, respectively.

#### Prepulse inhibition of the acoustic startle response (PPI)

Naive rats received ACPC (100–400 mg/kg IP) 15 min before the test. In ketamine-disturbed conditions, ACPC (200–400 mg/kg, IP) and ketamine (10 mg/kg SC) were administered 15 and 5 min, respectively, before the test.

#### Five-choice serial reaction time task (5-CSRTT)

ACPC (200–400 mg/kg, IP) and MK-801 (12.5–50 μg/kg, SC) were administered 30 and 10 min before the test, respectively.

### Data analysis

Data were analyzed using IBM/SPSS 21 for Windows. The alpha value was set at *P* < 0.05. The homogeneity of variance was measured with Levene’s test.

#### Novel object recognition test (NOR)

Discrimination index (DI) data were analyzed using one-way ANOVAs with drug dose as between-subject factor. Sidak’s test was used as a post hoc test (Howell 1997).

#### Attentional set-shifting test (ASST)

The number of trials required to achieve the criterion of six consecutive correct responses was recorded for each rat and for each discrimination phase. The data were assessed by two-way ANOVA with ACPC dose as a between-subject factor and discrimination phase (SD, CD, Rev 1, ID, etc.) as a within-subject factor. Sidak’s test was used as a post hoc test.

#### Spontaneous and PCP-induced hyperactivity

The activity data collected every 5 min are presented as area under curve of the total distance traveled, in centimeter. The measurement of the first 30 min indicates ACPC- or clozapine-induced effects on spontaneous locomotor activity. The second period (measured in the same animals 15–45 min following PCP administration) indicates drug-induced alteration of PCP-induced hyperactivity (Gleason and Shannon 1997). Separate ANOVAs followed by Dunnett’s multiple comparison tests were used to assess the effects of compounds on activity.

#### Conditioned avoidance response (CAR)

Separate one-way ANOVAs, followed by Tukey’s post hoc tests, were used to assess the effects of ACPC and clozapine on the percentage of conditioned avoidance responding and of escape failures.

#### Prepulse inhibition of the acoustic startle response (PPI)

The mean response amplitude for pulse-alone [P] and prepulse + pulse [PP] trials was computed for each rat, and PPI was determined according to the formula: PPI (%) = [(P−PP)/P] × 100. Percentage PPI data were analyzed using two-way ANOVAs with the between-subject factors of drug treatment and prepulse intensity as a within-subject factor followed by Fisher's protected LSD. Pulse amplitude values, (calculated as the average response to all of the pulse-alone trials) were subjected to one-way ANOVAs.

#### Five-choice serial reaction time task (5-CSRTT)

The following parameters were recorded in each session: percent of accuracy (number of correct responses divided by the sum of correct and incorrect responses × 100), number of premature responses (total number of responses performed during the ITIs), omissions (total number of trials omitted during a 100-trial session), correct response latency (time from the stimulus onset to a correct response), reward latency (time from a correct response to the retrieval of food from the magazine), as well as perseverant responses (total number of responses emitted after a correct response has been made). The data regarding the accuracy (the main measure of performance) and the number of premature responses (the main measure of impulsivity) are presented on Fig. 6; the other data are presented in Table 3. Separate one-way ANOVAs, followed by Tukey’s post hoc tests, were used to assess the effects of ACPC and MK-801 on the outcome of 5-CSRTT.

## Results

### Novel object recognition test (NOR)

**Fig. 1:**
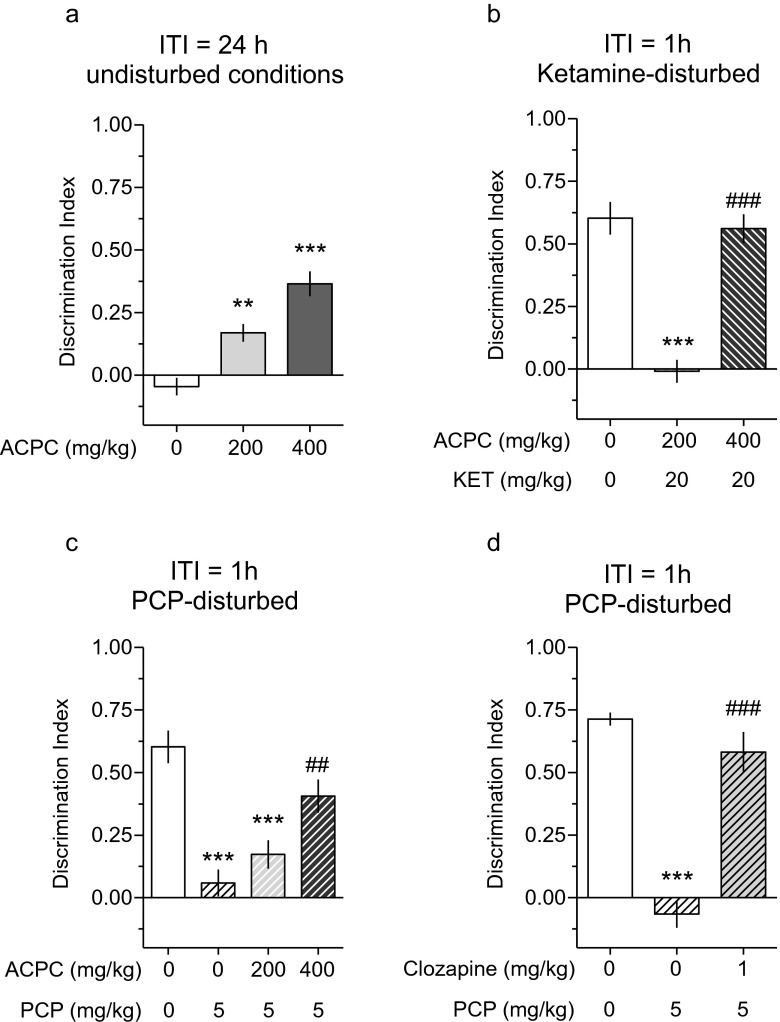
(**a**) ACPC improves object recognition memory in the novel object recognition (NOR) test at a long (24 h) inter-trial interval (ITI) in naive animals (**P < 0.01, ***P < 0.001 vs. vehicle; *N* = 10 rats per dose). (**b**) At a short (1 h) ITI, ketamine (20 mg/kg) disturbs object recognition memory (***P < 0.001 vs. vehicle) and 400 mg/kg of ACPC prevents ketamine-induced disturbance (^###^ *P* < 0.001 vs. vehicle + ketamine); *N* = 9–10 rats per dose. (**c**) At a short (1 h) ITI, PCP (5 mg/kg) disturbs object recognition memory (***P < 0.001 vs. vehicle) and 400 mg/kg, but not 200 mg/kg of ACPC, prevents PCP-induced disturbance (##*P* < 0.01 vs. vehicle + PCP); *N* = 8–10 rats per dose. (**d**) A similar procognitive effect of 1 mg/kg of clozapine, which at a short (1 h) ITI, prevents from PCP-induced disturbance (###*P* < 0.001 vs. vehicle + PCP); *N* = 9–11 rats per dose. Values represent the mean (±SEM) of discrimination index

As shown in Fig. 1a, ACPC dose-dependently improved object recognition memory in naive rats at the long (24 h) inter-trial interval: *F*(2.27) = 25.35, *P* < 0.001. At 1 h inter-trial interval, ketamine (20 mg/kg) produced an impairment of object recognition memory, which was alleviated by 400 mg/kg of ACPC: *F*(2.26) = 41.81, *P* < 0.001 (Fig. 1b). At 200 and 400 mg/kg, ACPC also prevented PCP-induced impairment of object recognition memory: *F*(3.33) = 14.82, *P* < 0.001 (Fig. 1c). The “positive” control, clozapine (1 mg/kg), similarly prevented an impairment of object recognition memory in PCP-induced disturbed conditions: *F*(2.26) = 42.76, *P* < 0.001 (Fig. 1d). Table 1 shows that the compounds used did not affect the exploration of objects during familiarization phase (T1).

**Table 1:** The effects of treatments on the exploration of objects A1 and A2 used during test 1 in the novel object recognition test

Treatment (*N*)	Object A1 exploration (*s*)	Object A2 exploration (*s*)
ITI = 24 h, undisturbed conditions		
Vehicle (10)	12.66 ± 1.07	11.70 ± 1.13
ACPC 200 mg/kg (10)	10.84 ± 0.67	11.25 ± 1.20
ACPC 400 mg/kg (10)	11.31 ± 1.38	12.15 ± 1.08
Statistics of two-way mixed-design ANOVA	Treatment: *F*(2.27) = 0.33; NS Object: *F*(1.27) = 0.02; NS Treatment × object: *F*(2.27) = 0.88; NS	
ITI = 1 h, ketamine disturbed		
Vehicle (10)	8.37 ± 0.78	9.43 ± 0.62
VEH + KET 20 mg/kg (9)	10.38 ± 0.95	12.09 ± 1.20
ACPC 400 + KET 20 mg/kg (10)	9.15 ± 0.68	10.58 ± 1.06
Statistics of two-way mixed-design ANOVA	Treatment: *F*(2.26) = 2.50; NS Object: *F*(1.26) = 5.44; *P* < 0.05 Treatment × object: *F*(2.26) = 0.90; NS	
ITI = 1 h, PCP disturbed		
Vehicle (8)	10.83 ± 0.73	9.75 ± 0.59
VEH + PCP 5 mg/kg (9)	9.23 ± 1.13	9.61 ± 0.82
ACPC 200 + PCP 5 mg/kg (10)	8.85 ± 1.27	8.73 ± 1.09
ACPC 400 + PCP 5 mg/kg (10)	9.89 ± 1.46	10.01 ± 0.94
Statistics of two-way mixed-design ANOVA	Treatment: *F*(3.33) = 0.50; NS Object: *F*(1.33) = 0.08; NS Treatment × object: *F*(3.33) = 0.26; NS	
ITI = 1 h, PCP disturbed		
Vehicle (9)	8.76 ± 1.08	8.54 ± 0.52
VEH + PCP 5 mg/kg (9)	7.31 ± 1.00	7.60 ± 1.13
Clozapine 1 + PCP 5 mg/kg (11)	6.95 ± 1.18	9.08 ± 0.86
Statistics of two-way mixed-design ANOVA	Treatment: *F*(2.26) = 0.53; NS Object: *F*(1.26) = 1.03; NS Treatment × object: *F*(2.26) = 1.05; NS	

### Attentional set-shifting test (ASST)

**Fig. 2:**
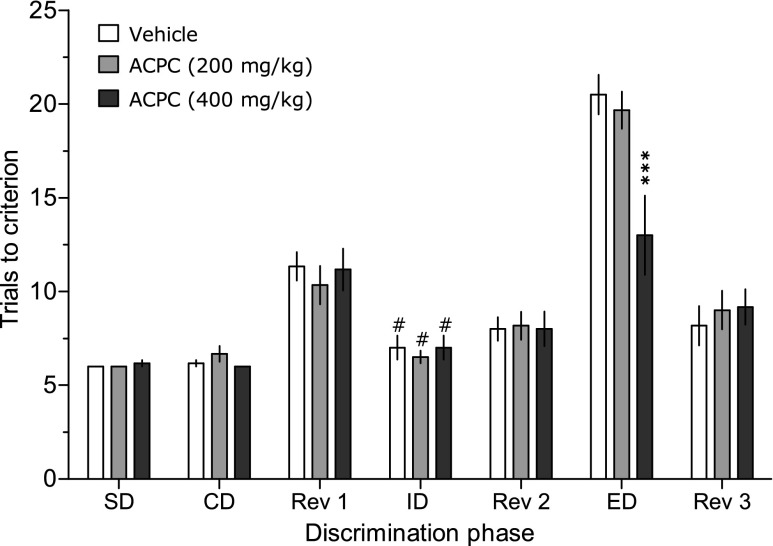
ACPC enhances cognitive flexibility in the attentional set-shifting test (ASST) in otherwise untreated rats at the dose of 400 but not 200 mg/kg. ***P < 0.001 vs. vehicle at the ED phase, #*P* < 0.01 vs. respective ED phase, *N* = 6 rats per dose. Values represent the mean (±SEM) number of trials to a given criterion

Figure 2 shows that the animals required more trials to the criterion at the ED phase than at the ID phase, suggesting that the presently used experimental conditions allowed for building an attentional set. ACPC reduced the number of trials to criterion at the ED phase (Fig. 2) at the dose of 400 but not 200 mg/kg, suggesting an improvement of cognitive flexibility. Two-way ANOVA demonstrated a significant interaction between the dose and the phase of discrimination: *F*(12.90) = 3.85, *P* < 0.001.

### Spontaneous and PCP-induced hyperactivity

**Fig. 3:**
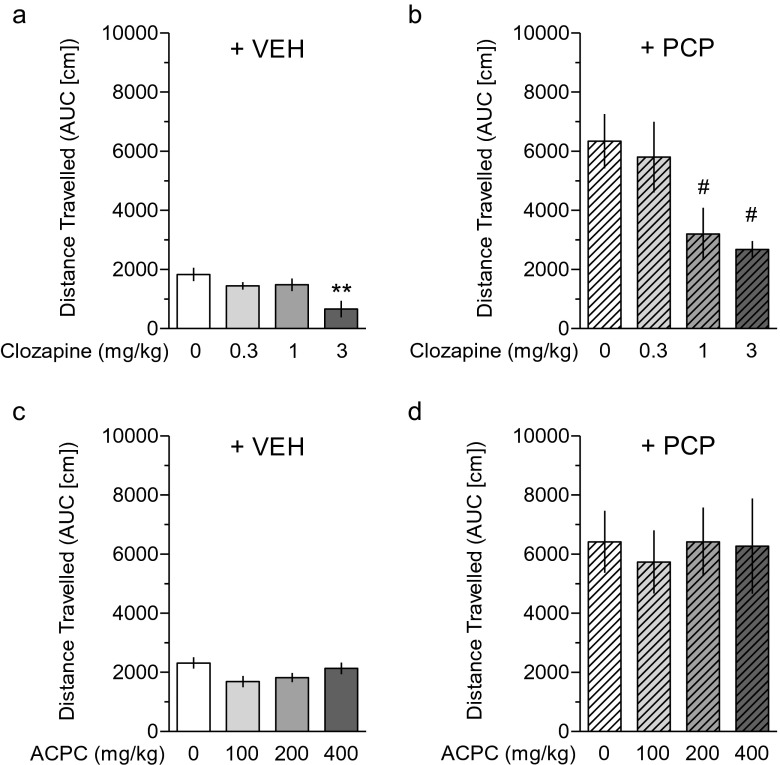
In contrast to clozapine, which inhibited PCP-induced hyperactivity (**b)** at a dose lower than that producing sedation (**a)**, ACPC affected neither spontaneous activity (**c)** nor PCP-induced hyperactivity (**d)**. Values represent the mean (±SEM) distance traveled, expressed as the area under the curve (AUC), in centimeter. Spontaneous activity was measured for the first 30 min following drug administration. PCP-induced hyperactivity was also measured for 30 min, starting 15 min after PCP administration. **P < 0.01 vs. vehicle control, #*P* < 0.05 vs. PCP. *N* = 8–9 rats per dose (clozapine study) and 8–16 rats per dose (ACPC study)

Figure 3a demonstrates that clozapine inhibited spontaneous activity at the dose of 3 mg/kg (*F*(3.30) = 4.94, *P* < 0.005). In addition, clozapine attenuated PCP-induced hyperactivity already at the dose of 1 mg/kg (*F*(3.30) = 4.23, *P* < 0.025, Fig. 3b), a characteristic for antipsychotic-like activity. In contrast, ACPC affected neither spontaneous activity (*F*(3.39) = 0.1, Fig. 3c) nor PCP-induced hyperactivity (*F*(3.39) < 1, Fig. 3d).

### Conditioned avoidance response (CAR)

**Fig. 4:**
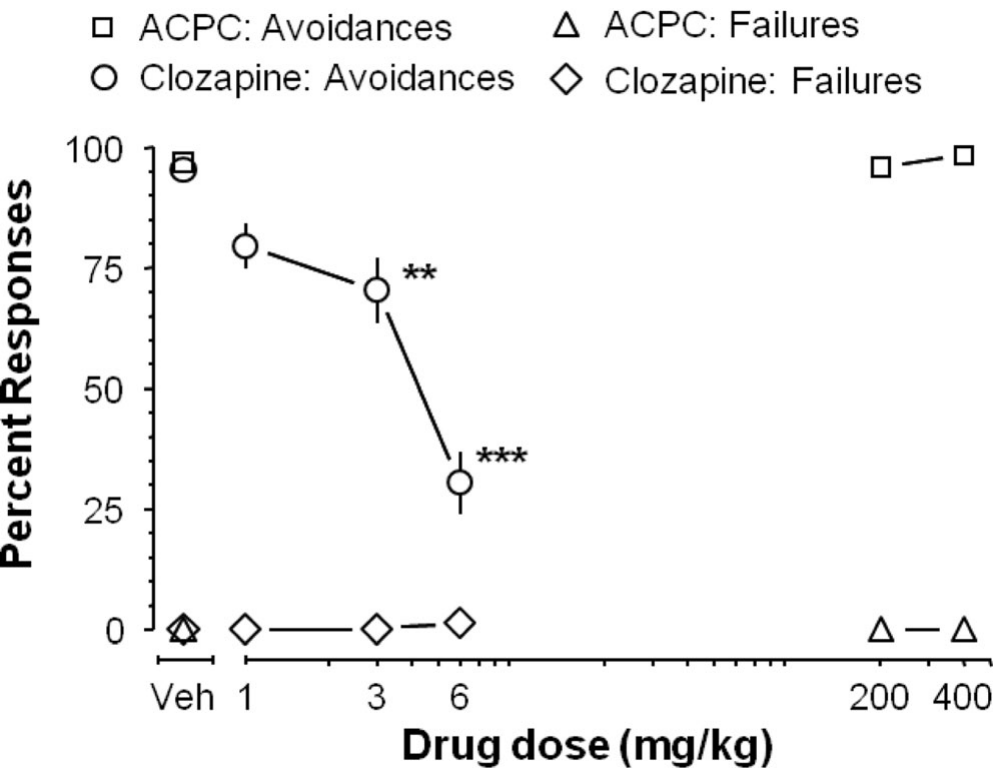
In the conditioned avoidance response (CAR) test, ACPC affected neither avoidance responses nor escape failures. In contrast, clozapine (3 and 6, but not 1 mg/kg) selectively inhibited avoidance responses without affecting escape failures, indicating an antipsychotic-like effect. Values represent the mean (±SEM) percentage of avoidance responses and escape failures. **P < 0.01, ***P < 0.001 vs. vehicle control. *N* = 5–8 rats per dose (ACPC study) and 10–11 rats per dose (clozapine study)

ACPC did not affect conditioned avoidance responding: *F*(2.16) = 0.49, NS. In contrast, clozapine (3 and 6 mg/kg) reduced avoidance responses, indicative of antipsychotic-like efficacy, *F*(3.42) = 26.85, *P* < 0.001. Clozapine did not affect escape failures (*F*(3.42) = 1.10, NS) indicating that at these doses, it does not produce sedation or motor side effects (Fig. 4).

### Prepulse inhibition of the acoustic startle response (PPI)

**Fig. 5:**
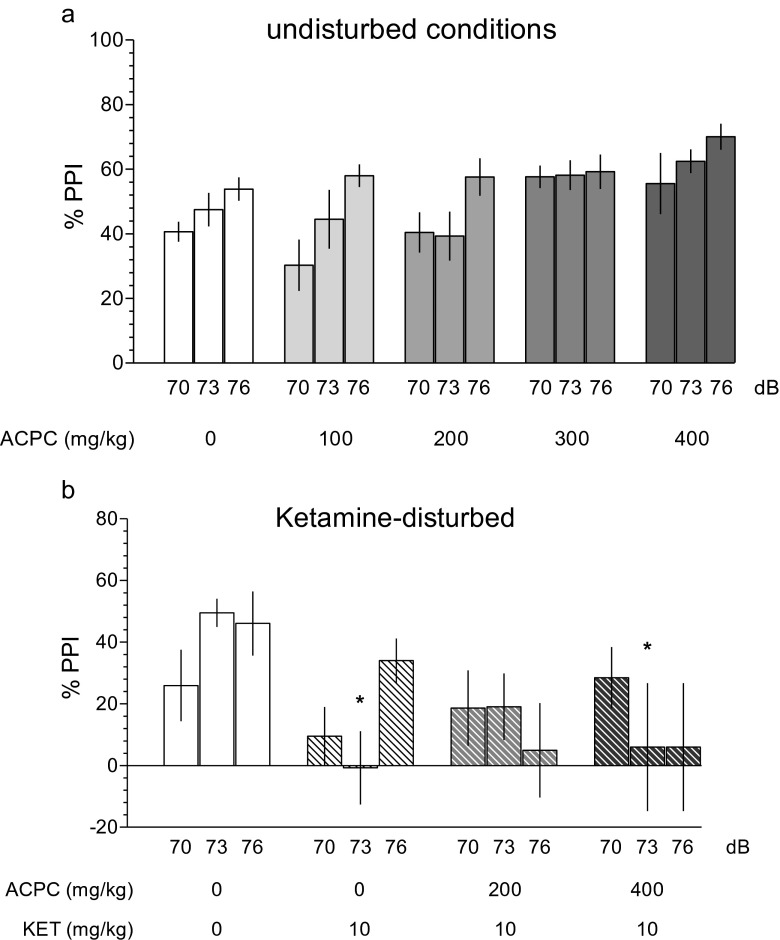
In the prepulse inhibition of the acoustic startle response (PPI) test, ACPC affected prepulse inhibition response neither in (**a**) naive animals, suggesting the lack of psychotomimetic effects, nor in (**b**) ketamine-induced PPI disturbed conditions, suggesting the lack of antipsychotic-like effects. Values represent the mean (±SEM) percentage of prepulse inhibition. *P < 0.05, vs. vehicle control at a given prepulse intensity expressed in decibel. *N* = 4–15 rats per dose (naive animals) and 4–11 rats per dose (ketamine-disturbed conditions)

ACPC did not affect PPI in naive animals (Fig. 5a, *F*(4.33) = 2.12, NS). There was no interaction between dose and prepulse intensity: *F*(8.66) = 1.80, NS, but, as expected, a significant effect of prepulse intensity: *F*(2.66) = 17.90, *P* < 0.001. One-way ANOVA showed significant differences in the pulse amplitude: *F*(4.33) = 2.80, *P* < 0.05, and ACPC at only the single dose of 200 mg/kg increased startle amplitude as compared with vehicle (Table 2).

**Table 2:** Effects of ACPC, ketamine, and combinations of these compounds on the startle amplitude evoked by a 120-dB pulse tone in rats

Dose of ACPC	Mean ± SEM (*N*) startle amplitude in naive rats
0	519.01 ± 75.54 (15)
100	429.87 ± 68.01 (6)
200	911.19 ± 171.42 (7)*
300	580.37 ± 82.44 (6)
400	688.72 ± 98.58 (4)
Treatment	Mean ± SEM (*N*) startle amplitude in KET-treated rats
VEH-VEH	557.70 ± 114.42 (6)
KET-VEH	618.38 ± 76.95 (11)
KET-ACPC 200	701.8 ± 138.87 (5)
KET-ACPC 400	763.08 ± 137.65 (6)

*P < 0.05 vs. vehicle

Figure 5b shows that ACPC did not affect PPI under ketamine-disturbed conditions. Two-way ANOVA showed no significant treatment effect (*F*(3.24) = 1.43, NS and prepulse intensity: *F*(2.48) = 0.25). The significant interaction between treatment and prepulse intensity: *F*(6.48) = 3.57, *P* < 0.01 allowed for performing a post hoc test, which revealed that at an intensity of 73 dB, both vehicle + ketamine as well as 400 mg/kg of ACPC + ketamine-disrupted PPI, suggesting a lack of antipsychotic-like activity of ACPC. One-way ANOVA showed no significant effects of treatment on pulse amplitude: *F*(3.24) = 0.6, NS (Table 2).

### Five-choice serial reaction time task (5-CSRTT)

**Fig. 6:**
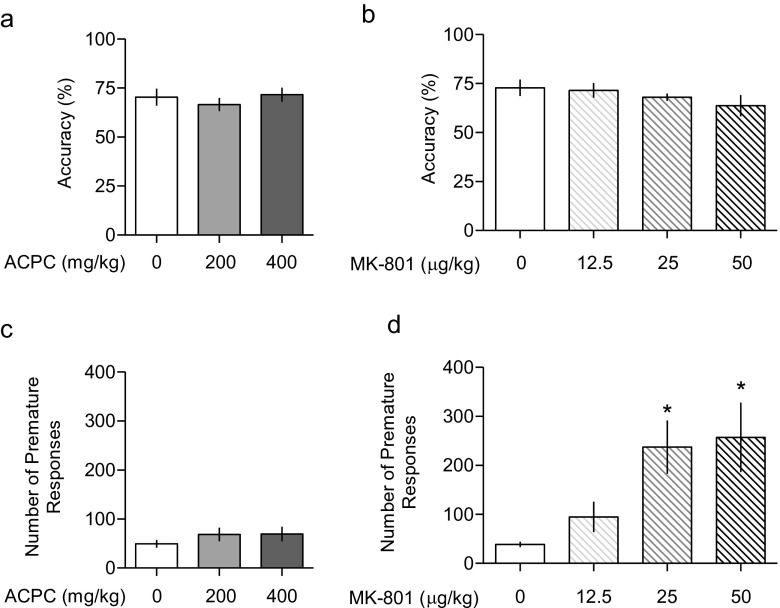
Neither (**a**) ACPC nor (**b**) MK-801 affected the accuracy in the five-choice serial reaction time task (5-CSRTT). However, while (**c**) ACPC did not affect the number of premature responses, a measure of impulsivity, (**d**) MK-801 dose-dependently increased this measure. Values represent the mean (±SEM) percentage of premature responses and number of premature responses. *P < 0.05, vs. vehicle. *N* = 9−12 rats per dose (ACPC study) and 7–8 rats per dose (MK-801 study)

ACPC affected neither the percentage of accuracy: *F*(2.29) = 0.52, Fig. 6a nor the number of premature responses: *F*(2.29) = 0.60, Fig. 6c. Table 3 shows that ACPC did not affect omissions, correct response latency, reward latency, or perseverant responses. While MK-801 at relatively low (25–50 μg/kg) doses did not affect accuracy: *F*(3.27) = 1.03, Fig. 6b, as expected (Higgins et al. 2003) it dose-dependently increased the number of premature responses, a measure of impulsivity: *F*(3.27) = 5.48, *P* < 0.01, Fig. 6d. Consistent with these effects, treatment with MK-801 reduced correct response latency and reward latency, but did not affect the number of omissions and perseverant responses (Table 3).

**Table 3:** Effects of ACPC and MK-801 on omissions (total number of trials omitted during a 100-trial session), correct response latency (time from the stimulus onset to a correct response), reward latency (time from a correct response to the retrieval of food from the magazine), and perseverant responses (total number of responses emitted after a correct response has been made) in the five-choice serial reaction time task. Values represent the mean (±SEM). *N* = 9–12 rats per dose (ACPC study) and 7–8 rats per dose (MK-801 study)

Treatment	Omissions (*n*)	Latency correct (*s*)	Latency reward (*s*)	Perseverant (*n*)
Vehicle	12.9 ± 2.5	0.96 ± 0.07	1.73 ± 0.08	1.6 ± 0.7
ACPC 200 mg/kg	10.9 ± 1.6	0.99 ± 0.08	1.56 ± 0.13	1.8 ± 0.8
ACPC 400 mg/kg	11.8 ± 1.6	0.81 ± 0.07	1.53 ± 0.11	2.0 ± 0.9
	*F*(2.29) = 0.27	*F*(2.29) = 1.85	*F*(2.29) = 0.84	*F*(2.29) = 0.07
	NS	NS	NS	NS
Vehicle	13.5 ± 2.6	0.93 ± 0.04	1.80 ± 0.1	3.6 ± 0.8
MK-801 12 μg/kg	8.1 ± 1.8	0.75 ± 0.03*	1.46 ± 0.08*	2.8 ± 1.4
MK-801 25 μg/kg	13.6 ± 2.0	0.78 ± 0.04*	1.47 ± 0.07*	2.3 ± 0.8
MK-801 50 μg/kg	18.0 ± 4.9	0.84 ± 0.04	1.39 ± 0.06*	1.1 ± 0.7
	*F*(3.27) = 1.81	*F*(3.27) = 4.25	*F*(3.27) = 4.91	*F*(3.27) = 1.14
	NS	*P* < 0.05	*P* < 0.01	NS

*P < 0.05, vs. vehicle

## Discussion

The present study demonstrates that ACPC, a partial agonist at the strychnine-insensitive glycine receptors (Marvizon et al. 1989), facilitates object recognition learning in the novel object recognition test in both the presence and absence of NMDAR antagonists, and enhances cognitive flexibility in the attentional set-shifting task. However, ACPC produced no measurable effects in PCP-induced hyperactivity, conditioned avoidance response, and prepulse inhibition tests, suggesting a lack of antipsychotic-like activity. Like MK-801, ACPC did not affect accuracy in the five-choice serial reaction time task, but in contrast to very low doses of MK-801 (Higgins et al. 2003), no increase in impulsivity was observed. The latter findings strongly suggest prominent differences in the pharmacological profile of ACPC compared to NMDA channel blockers. Thus, despite the functional antagonist properties of ACPC documented in multiple studies, it did not disturb object recognition learning like PCP and ketamine (Fig. 1), did not disturb prepulse response like ketamine (Fig. 5), and did not affect impulsivity like MK-801 (Fig. 6).

### Cognition

While an impairment of cognitive flexibility due to NMDAR blockade has been broadly reported (Stefani et al. 2003; Egerton et al. 2005; Nikiforuk et al. 2010), much less is known about the effects of agents enhancing NMDAR-mediated responses on ASST performance.

Preclinical studies with glycine site ligands demonstrated procognitive actions of the glycine agonist D-serine (Duffy et al. 2008) and the partial agonist ACPC (Popik and Rygielska 1999) in the Morris water maze, as well as the partial agonist D-cycloserine in the radial maze (Pussinen and Sirvio 1999). In addition, the GlyT1 inhibitor SSR-504734 facilitated working memory in a continuous delayed alternation task in mice (Singer et al. 2009). Perhaps more relevant to the present work are the findings of Shimazaki et al. (2010) demonstrating that the GlyT1 inhibitor, NSFP, improved MK-801-disrupted social memory (Shimazaki et al. 2010) and of Karasawa et al. (2008), who reported that D-serine and NFPS improved learning in the NOR test in conditions disturbed by MK-801 administration. The present data with ACPC demonstrating a similar alleviation of learning disturbances induced by PCP and ketamine in the novel object recognition task, confirm these findings and appear as a logical consequence of its partial agonistic activity at the glycine/NMDA sites.

The present results with ACPC also extend, and are consistent with, our recent findings with a GlyT1 inhibitor (Nikiforuk et al. 2011), demonstrating that increased NMDAR tone may facilitate cognitive flexibility, which is compromised in schizophrenia (Lewis and Gonzalez-Burgos 2008). Because NMDA channel blockers produce symptoms of psychoses (Morris et al. 2005) and NMDARs are hypothesized to be hypoactive in schizophrenia (Coyle et al. 2003), the procognitive effects of ACPC suggest a potential for this type of compound in treating the cognitive deficits associated with psychoses.

### Antipsychotic-like effects

The rationale for studying the potential antipsychotic-like effects of a glycine site partial agonist was based on reports indicating similar actions of glycine full agonists like D-serine, the partial agonist D-cycloserine and GlyT1 inhibitors, already in clinical trials; for a review, see (Harvey and Yee 2013). However, despite using three different tests, we were unable to demonstrate antipsychotic-like effects of ACPC.

Karcz-Kubicha et al. (1999) reported that ACPC (600 mg/kg) enhanced PCP-induced hyperactivity; the effects of lower doses were not investigated. While the lack of efficacy of ACPC in affecting PCP-induced hyperactivity reported here could be due to insufficient dosing, doses ≤400 mg/kg of ACPC produced a number of procognitive effects (Figs. 1 and 2; as well as other therapeutic (antidepressant-like, neuroprotective, anxiolytic) actions (see the “Introduction” section)).

In apparent contrast to Karcz-Kubicha et al. (1999) and the present findings, an enhancement of glycine site function reduces behavioral manifestations associated with NMDA receptor blockade. For instance, glycine transporter inhibitors reversed or attenuated NMDAR antagonist-induced deficits in PPI (Lipina et al. 2005; Depoortere et al. 2005; Boulay et al. 2008), suggesting that stimulation of glycine/NMDA sites leads to antipsychotic-like effects. This is consistent with human data showing that ketamine-induced schizophrenia-like symptoms were ameliorated by GlyT1 inhibitor Org 25935 in healthy humans (D'Souza et al. 2012). In light of these findings, the lack of efficacy of ACPC in the tests predictive of antipsychotic action could be viewed as unexpected. It should be noted, however, that the present results in the PPI test should be treated with caution. While a significant ketamine-induced disruption of PPI was observed (Fig. 5b), the effects of ketamine were modest compared to other reports (Swerdlow et al. 1998). ACPC at the highest doses of 300 and 400 mg/kg per se appeared to increase PPI (Fig. 5a), but the statistical analysis did not find these increases significant. Nonetheless, such a purportedly antipsychotic-like effect would not be consistent with the data indicating that neither clozapine, haloperidol, nor chlorpromazine per se increased the PPI response (Swerdlow et al. 1998). Finally, ACPC at a single dose (200 mg/kg) increased startle amplitude, but this effect was neither related to the dose nor consistent with a purported antipsychotic-like action, as startle amplitude is increased by psychotomimetic compounds like MK-801 (Bakshi et al. 1994).

### ACPC: from an agonist to a functional antagonist at the glycine/NMDA sites

In the presence of saturating concentrations of glutamate, the efficacy of ACPC is high, ~90 % relative to glycine (Marvizon et al. 1989; Karcz-Kubicha et al. 1997). However, ACPC was reported to exhibit only 60 % of the glycine’s activity in enhancing [^3^H]MK-801 binding in the nominal absence of glutamate (that is, using well-washed brain membranes; (Marvizon et al. 1989)) suggesting a functional antagonist profile dependent upon ambient concentrations of both glutamate and glycine.

These findings may have important therapeutic implications because they suggest that when glutamate concentrations are high, as in learning and memory tasks (Danysz and Parsons 1998), ACPC could display an agonist-like profile, enhancing cognitive processes. In contrast, in the hypo-glutamatergic states associated with psychoses (Coyle et al. 2003), ACPC behaves like a functional glycine receptor antagonist and exhibits no antipsychotic-like efficacy.

Clinical studies are consistent with this hypothesis. Thus, while an addition of the partial agonist, D-cycloserine, to risperidone in schizophrenic patients reduced negative symptoms (Evins et al. 2002), meta-analyses showed that glycine site full agonists are more effective than D-cycloserine in improving the overall psychopathology in schizophrenic patients receiving antipsychotic drugs (Heresco-Levy and Javitt 2004; Tuominen et al. 2005; Tsai and Lin 2010). Nonetheless, the recent reports indicating that GlyT1 inhibitors do not improve clinical symptoms of schizophrenia in large, phase III trials (Kingwell 2014) raise serious questions about the viability of glutamate-based approaches to treat schizophrenia. At a minimum, these discrepant findings indicate that not all methods of elevating glycinergic tone produce equivalent results in the clinic. In essence, this raises the bar for preclinical evidence sufficient to trigger clinical studies. Nonetheless, like NMDAR antagonists (Layer et al. 1998), D-cycloserine at relatively high doses, suggestive of NMDAR antagonistic actions, was recently reported to produce an antidepressant effect in treatment-resistant depressed patients (Heresco-Levy et al. 2013). These clinical observations reveal the potential for glycine site partial agonists to mimic the pharmacological actions of both glycine site agonists and antagonists, which is consistent with the present demonstration that ACPC possesses procognitive, but not antipsychotic-like efficacy. Thus, we postulate that a compound with a neurochemical profile like that of ACPC may represent a useful pharmacological approach for cognitive enhancement in domains critically affected in schizophrenia, but would not affect negative or positive symptoms of the psychoses.
